# Protective Mechanisms of Vaginal Lactobacilli against Sexually Transmitted Viral Infections

**DOI:** 10.3390/ijms25179168

**Published:** 2024-08-23

**Authors:** Elisa Avitabile, Laura Menotti, Vanessa Croatti, Barbara Giordani, Carola Parolin, Beatrice Vitali

**Affiliations:** Department of Pharmacy and Biotechnology, University of Bologna, 40127 Bologna, Italy; elisa.avitabile@unibo.it (E.A.); laura.menotti@unibo.it (L.M.); vanessa.croatti2@unibo.it (V.C.); barbara.giordani4@unibo.it (B.G.); b.vitali@unibo.it (B.V.)

**Keywords:** vaginal health, sexually transmitted infections, vaginal microbiome, lactobacilli, sexually transmitted viruses, HPV, HIV, HSV

## Abstract

The healthy cervicovaginal microbiota is dominated by various *Lactobacillus* species, which support a condition of eubiosis. Among their many functions, vaginal lactobacilli contribute to the maintenance of an acidic pH, produce antimicrobial compounds, and modulate the host immune response to protect against vaginal bacterial and fungal infections. Increasing evidence suggests that these beneficial bacteria may also confer protection against sexually transmitted infections (STIs) caused by viruses such as human papillomavirus (HPV), human immunodeficiency virus (HIV) and herpes simplex virus (HSV). Viral STIs pose a substantial public health burden globally, causing a range of infectious diseases with potentially severe consequences. Understanding the molecular mechanisms by which lactobacilli exert their protective effects against viral STIs is paramount for the development of novel preventive and therapeutic strategies. This review aims to provide more recent insights into the intricate interactions between lactobacilli and viral STIs, exploring their impact on the vaginal microenvironment, host immune response, viral infectivity and pathogenesis, and highlighting their potential implications for public health interventions and clinical management strategies.

## 1. Vaginal Microbiota in Health and Disease

The human body is home to diverse microbial communities, colonizing different niches directly linked or not to the external environment and playing elementary roles in health and diseases [1]. The assorted population of bacteria, viruses, fungi, and other unicellular organisms living in or on humans is defined as “microbiota”. The collection of all the genes within these microorganisms is known as a “microbiome” [2]. Next Generation Sequencing (NGS) techniques based on the 16S rRNA gene permit in-depth study of microbial community structures, providing the taxonomic identity of the microbiome and expanding our understanding of the pathophysiology underlying various diseases affecting different body systems [3,4]. According to a re-visited definition, the microbiome is not only the collection of genes but also a “theatre of activity”, including the structural elements, metabolites/signal molecules, and the surrounding environmental conditions [5]. Indeed, new approaches, such as metagenomic and metatranscriptomic, aimed at investigating the gene content and expression of microbial communities, are emerging in perspective to elucidate not only the composition but also the functional role of the microbiota in human health and disease [6,7].

### 1.1. The Microbiome of the Female Reproductive Tract

Within the human microbiome, the female reproductive tract houses 9% of the total microbial population of the entire body [8]. Distinct microbial communities exist throughout the female reproductive tract, which can be divided into two parts: (i) the upper tract comprises the endocervix, endometrium, uterine cavity, fallopian tubes, ovary, peritoneal fluid, and, during pregnancy, placenta; (ii) the lower tract, known as cervicovaginal tract, comprises the vagina and the cervix together. Bacterial colonization gradually decreases from the lower reproductive tract to the upper one. Uterine bacteria are estimated to be about 10,000 times lesser than that of the cervicovagina, and the most dominant ones are *Prevotella* spp., *Lactobacillus iners*, and *Lactobacillus crispatus* [9].

The microbiota of the cervicovaginal tract resides in and on the epithelium’s outermost layer [10], and its composition changes during the entire female lifecycle (from childhood up to menopause), being influenced by hormone levels, age, genetics and external factors like diet, sexual behavior, hygiene habits and antibiotic consumption [11,12]. The pre-pubescent cervicovaginal microbiota includes different microbial communities, with the dominancy of anaerobes, i.e., the *Enterobacteriaceae* and/or *Staphylococcacee* family [13]. In reproductive-aged women, the elevated level of estrogen, glycogen, and the thick vaginal epithelium ensure the optimal conditions for the colonization of *Lactobacillus* spp., which contribute to the acidification of the cervicovaginal region by producing principally lactic acid and some other organic acids [14]. During the menopausal stage, the estrogen level drops, and the vaginal epithelium becomes thinner with low levels of glycogen, thus resulting in a decline in the lactobacilli population, increased vaginal pH (>5) and greater susceptibility to infections [15]. In pregnancy, the absence of menses, an increased level of placental estrogen and a thicker vaginal mucosa stuffed with glycogen lead to an increase in lactobacilli that, in turn, lower the vaginal pH (<4.5) and the chances of contracting infections during pregnancy are reduced [16].

Traditionally, the neonate’s microbiome, including the cervicovaginal one, was thought to be established only from birth. However, new evidence suggests the presence of microorganisms in the fetal gut before birth, known as the ‘prenatal’ microbiota. This hypothesis proposes that microbial colonization may occur in utero, with maternal microbes from the oral, gut, and vaginal regions potentially reaching the fetus via the bloodstream or vaginal tract. As a result, the maternal microbiome during pregnancy could influence the neonate’s vaginal microbiome even before birth, though further research is needed to clarify this hypothesis [17].

### 1.2. Community State Types (CSTs)

With the emergence of NGS techniques, the vaginal microbiome of women in their reproductive age has been categorized into five primary community state types (CSTs) based on community composition and abundance, determined at the taxonomic level [18]. Four CSTs (I, II, III, and V) display lower diversity with the dominance of a single *Lactobacillus* species and are associated with reproductive health. Specifically, these healthy CSTs are characterized by the dominance of *Lactobacillus crispatus* (CST I), *Lactobacillus gasseri* (CST II), *Lactobacillus iners* (CST III) and *Lactobacillus jensenii* (CST V). In contrast, CST IV is typically devoid of *Lactobacillus* spp. and enriched with obligate anaerobic species, such as *Gardnerella* spp., *Megasphaera* spp., *Sneathia* spp. and *Prevotella* spp. This higher species diversity and non-*Lactobacillus* dominance is associated with increased risk for a variety of health problems, including gynecological and pregnancy complications [19]. In addition, metagenomic sequencing revealed strain-level variation in vaginal community composition, identifying 27 metagenomic CSTs that differentially correlate with disease symptoms [7]. However, understanding of vaginal microbiome variations across geographical and ethnic groups remains limited. It was reported that *Lactobacillus*-enriched CSTs (groups I, II, III, and V) were more frequently retrieved in reproductive-aged White and Asian women, compared to Hispanic and Black ones, mostly characterized by CST IV [18]. Subsequently, it was found that CST I is more likely to occur in White or Asian women, while Black women mainly host CST IV (A and B) [20]. Thus, *Lactobacillus*-dominated CSTs have traditionally become synonymous with healthy status, and White (and Asian) women were taken as the gold standard population in taxonomic studies of the vaginal microbiome composition and even of probiotics efficacy [21]. A definitive picture of the representation of vaginal CSTs over ethnic groups has yet to be determined, and the factors driving these differences are likely to be multifaceted, although recognizing these differences would be essential for comprehensive women’s health care.

### 1.3. Vaginal Eubiosis and Dysbiosis

Given that a cervicovaginal microbiota dominated by the *Lactobacillus* genus (10^7^–10^9^ lactobacilli/gram of vaginal fluid) is linked to a healthy eubiotic state [8], lactobacilli displacement by diverse bacterial populations has been associated with multiple gynecological complications broadly known as vaginal “dysbiosis”.

The most frequent type of cervicovaginal dysbiosis is bacterial vaginosis (BV), a polymicrobial clinical syndrome of reproductive-aged women characterized by a shift in the vaginal communities from the protective *Lactobacillus* species to facultative anaerobic bacteria (*Gardnerella vaginalis*) and strict anaerobic bacteria, i.e., *Prevotella* spp., *Fannyhessea vaginae* (formerly known as *Atopobium vaginae*), *Sneathia* spp., *Megasphaera* spp., etc. [22]. The changes in the bacterial communities occurring during BV result in significant alterations in the vaginal metabolome; in particular, the consumption of lactic acid, used for energy metabolism, and the production of amino compounds have been registered, with a consequent increase in vaginal pH (>4.5) [23,24,25]. BV condition enhances the risk of sexually transmitted infections (STIs), such as human papillomavirus (HPV), human immunodeficiency virus (HIV), *Trichomonas vaginalis*, *Chlamydia trachomatis* and *Neisseria gonorrhoeae* [26]. Furthermore, BV has been associated with complications in pregnancy, adverse effects on newborns, chorioamnionitis, premature deliveries, pelvic inflammatory disease, fetal loss, cuff cellulitis, postpartum endometritis, cervicitis and an increased risk of genitourinary infections [27].

Vulvovaginal candidiasis (VVC) is considered the second most prevailing dysbiosis after BV. Most VVC cases (75–90%) are attributable to *Candida albicans*, but recently, *Candida* non-*albicans* species infections have been increasingly diagnosed. The insurgence of VVC is related to the expression of *Candida* virulence factors, like morphologic switching and biofilm formation. Germ tubes, pseudohyphae, and hyphae promote *Candida* tissue invasion, while biofilms increase persistence with the aggravating circumstance of being resistant to antifungal drugs and host immune response [28]. Many studies have dealt with the ability of vaginal lactobacilli and their derivatives to reduce *Candida* spp. growth and virulence factors, highlighting the importance of healthy microbiota in preventing *Candida* infection [29,30,31].

## 2. Vaginal Lactobacilli: Physiological Roles and Molecular Mechanisms against Pathogens

**Figure 1:**
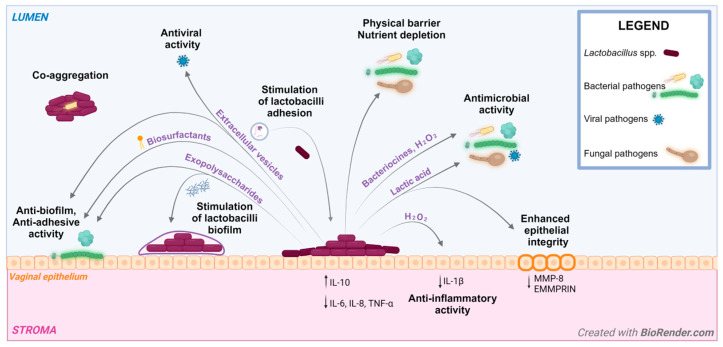
Schematic representation of the protective mechanisms of vaginal lactobacilli against pathogens.

The protective role of lactobacilli in the vaginal environment is attributed to a series of physiological mechanisms occurring between different actors: the lactobacilli cells and/or their derivatives, the microbial community and the host itself [32]. The major mechanisms are represented and summarized in Figure 1.

### 2.1. Mechanisms Relying on Metabolites Produced by Lactobacilli

The main health benefit provided by lactobacilli is the production of lactic acid through their anaerobic metabolism [33]. During the reproductive years, high estrogen levels ensure the presence of glycogen derivatives in the vagina that are converted by lactobacilli into L- and D-lactic acid, which helps maintain an acidic environment (pH ≤ 4), inhospitable for the proliferation of pathogens [34,35]. Lactic acid can impact various components of the vaginal microbiota, acting as an antimicrobial compound against bacterial, fungal and viral pathogens [31,36,37,38,39]. The most recognized biocidal mechanisms of lactic acid rely on its ability, or that of its released hydrogen ions, to alter the surface proteins of microorganisms and to penetrate lipid membranes, thereby acidifying the cytosol or disrupting microbial metabolism [40]. Moreover, lactic acid can regulate the host immune system against viral infections and maintain the integrity of host cells by triggering the autophagy processes [41,42]. Recently, it has been demonstrated that the specific isomer, either L- or D-, of lactic acid produced by lactobacilli can influence its activity [32]. The production of this acid by vaginal lactobacilli is species-dependent, with most *Lactobacillus* species capable of producing both isoforms, except for *L. iners*, which can only make the L-isomer [18,43]. D-lactate isoform from *L. crispatus*, *L. jensenii* and *L. gasseri* maintains tissue integrity, demonstrating a negative correlation with tissue degradative enzymes such as matrix metalloproteinase (MMP-8) and matrix metalloproteinase inducer (EMMPRIN) [44]. In addition, a higher ratio of L-lactate to D-lactate is positively correlated with tissue damage related to MMP-8 and EMMPRIN. This scenario is most observed when *L. iners* (CST IV) or *Gardnerella* spp. dominate the vaginal microbiota, elucidating why the prevalence of *L. iners* is more closely linked to the risk of vaginal dysbiosis and susceptibility to bacterial and viral sexually transmitted infections [45,46,47].

Hydrogen peroxide (H_2_O_2_) is a reactive compound normally produced by lactobacilli and released in the vaginal environment [48]. The production of H_2_O_2_ is typically related to some lactobacilli strains that present a facultative anaerobic metabolism, in which the presence of flavoproteins and the lack of heme groups induce the direct conversion of oxygen (O_2_) into H_2_O_2_. Strains belonging to *L. crispatus*, *L. jensenii* and *L. gasseri* are considered H_2_O_2_-producers, showing different amounts of production, while *L. iners*, *Lacticaseibacillus paracasei* and *Lactiplantibacillus plantarum* strains do not have the molecular pathway to produce it [47,49,50]. In vitro studies reported the microbicidal activity of H_2_O_2_ on *N. gonorrhoeae*, *C. albicans*, *Escherichia coli* and *Staphylococcus aureus*, as well as a virucidal effect against HIV-1 [51,52,53,54,55]. The biocide activity of H_2_O_2_ is related to its ability to pass through microorganisms’ membranes and react in the cytosol as an oxidant. In vivo, H_2_O_2_ can react with ferrous ions, generating a hydroxyl radical, which can rapidly oxidize cellular molecules such as nucleic acids, proteins, and lipids, thus resulting in mutagenetic and cytotoxic effects. This mechanism is highly efficient towards microorganisms that do not express H_2_O_2_ degradative enzymes like peroxidase or catalase (such as *Prevotella*, *Peptostreptococcus* and *Gardnerella*) [48,56]. At the same time, cervicovaginal cells present H_2_O_2_-neutralizing enzymes that ensure the protection of the cells from this molecule. Furthermore, it was demonstrated that exogenous H_2_O_2_ derived from lactobacilli can be used by epithelial and immune cells as a signaling molecule. Indeed, the abundance of lactobacilli H_2_O_2_-producers in the microbiota was related to a lower concentration of the pro-inflammatory cytokine IL-1β in the cervicovaginal fluid, indicating a possible immunosuppressive role of H_2_O_2_ [57]. In the gut context, it was demonstrated that *L. crispatus* uses H_2_O_2_ as a signal molecule to induce PPAR-γ activation in intestinal epithelial cells, thereby adjusting epithelial cell responsiveness to inflammatory stimuli [58]. Despite the abundance of lactobacilli in the vaginal environment, the concentration of H_2_O_2_ in the cervicovaginal fluid remains relatively low (23 ± 5 μM), primarily due to the physiological low levels of O_2_ in the vaginal lumen [37]. For this reason, it is still debatable the protective role of H_2_O_2_ produced by *Lactobacillus* species in in vivo conditions [59,60], although the capability of producing H_2_O_2_ is a recommended feature for the selection of probiotic strains [61]. Furthermore, vaginal lactobacilli produce small antimicrobial peptides such as bacteriocins or bacteriocin-like molecules that contribute to their protective effects against infections. These small molecules can form unstable or small-sized pores in cell membranes and/or inhibit enzymes involved in cellular metabolism, thus resulting in cytotoxicity [50]. Bacteriocins and bacteriocin-like molecules derived from vaginal strains belonging to *Ligilactobacillus salivarius*, *Limosilactobacillus fermentum*, *L. gasseri*, *L. crispatus*, *L. iners* and *L. jensenii* can inhibit the growth and/or biofilm formation of a plethora of opportunistic pathogens, such as *Enterococcus faecalis*, *Enterococcus faecium*, *G. vaginalis*, *S. aureus* and *Streptococcus agalactiae* [62,63,64,65,66]. A recent study reported that the vaginal microbiome is particularly enriched in species-specific gene clusters encoding for class II bacteriocins compared to other human niches. Bacteriocins activity may play a role in regulating microbial communities in the vagina, thus helping to maintain microbiome balance [67].

In addition, bacteriophages play a role in shaping the vaginal bacterial community. Interestingly, the *L. crispatus* M247 complete genome sequencing revealed the presence of Tn7088, a 14.1 kbp mobile genetic element, coding for 16 ORFs, including a class I bacteriocin, homologous to the listeriolysin S (lls) locus of *Listeria monocytogenes*. *L. crispatus* M247 genome also contains a siphovirus prophage ΦM247, coding for lysogenic and lytic cycle-related proteins, and three clustered regularly interspaced short palindromic repeats (CRISPRs) and 226 insertion sequences (ISs) [68]. These mobilizable and functional sequences, along with prophage integration, contribute to *Lactobacillus* genome plasticity and likely modulate the competitive interaction among different microbiota components, highlighting the complex interplay among host, microbiome and virome elements in the vaginal environment homeostasis [69].

Other complex metabolites/structures produced by vaginal lactobacilli that have been demonstrated to exert a protective mechanism against pathogens are biosurfactants (BS), exopolysaccharides (EPS) and extracellular vesicles (EVs). BS are amphiphilic compounds that can be attached to lactobacilli cell walls or released in the environment [70]. Thanks to their peculiar structure, BS can alter the chemistry of surfaces, thus modulating pathogens’ attachment and biofilm formation [71]. Moreover, they can interact with cell membranes and change their permeability with the loss of metabolites, leading to cell death [72]. BS from vaginal *L. paracasei* and *L. gasseri* inhibit *C. albicans* adhesion as well as biofilm formation [73,74]. Similarly, BS from *L. crispatus* BC1 inhibits *C. albicans* adhesion on human cervical HeLa cells and, in vivo, reduces leukocyte influx, thereby preventing mucosal damage [75]. Meanwhile, BS from *L. gasseri* strain BC9 reduces the biofilm formation of methicillin-resistant *S. aureus* isolates [76]. BS isolated from *L. jensenii* P6A and *L. gasseri* P65 reduce *E. coli*, *Staphylococcus saprophyticus, Enterobacter aerogenes* and *Candida* biofilms and the planktonic growth of *C. albicans* and *E. coli* [77]. Moreover, *L. crispatus* BC1 and *L. gasseri* BC10 produce BS able to reduce the growth of *N. gonorrhoeae* [78]. EPS are polymers mainly composed of sugars produced by bacteria that exhibit various functions based on their type, location, and complexity of the structure [79]. Recently, EPS released from different vaginal strains belonging to *L. crispatus* and *L. gasseri* species stimulated in a dose-dependent manner the biofilms of vaginal lactobacilli, including *Limosilactobacillus vaginalis*. In contrast, an inhibitory effect was demonstrated against the biofilm formation of *E. coli*, *Staphylococcus* spp., *Enterococcus* spp., *S. agalactiae* and *Candida* spp., indicating a role of these EPS in the modulation of biofilm structure within the vaginal microbiota [30]. The beneficial effect on lactobacilli biofilm formation can be ascribed to the fact that EPS can be a nutritional supplement to probiotic bacteria that express enzymes like α-galactosidase and β-galactosidase. The inhibitory effect towards pathogens can be related to the EPS structure that can modify the physical characteristics of microbial surfaces, thus reducing cell-to-cell interactions and/or inhibiting the expression of pathogens’ molecules involved in auto-aggregation processes. Moreover, EPS may exert a competitive inhibition of multivalent carbohydrate–protein interactions and shield host cell receptors available for pathogens’ recognition due to structural similarity [30]. As well as other Gram-positive bacteria, vaginal lactobacilli can release nanostructures, named EVs, that have been shown to impair HIV infection and pathogens’ adhesion. EVs from *L. crispatus* BC3 and *L. gasseri* BC12 reduce HIV-1 infection in ex vivo tissues by altering the exposure/expression of the glycoprotein gp120, responsible for the viral attachment/entry [80]. At the same time, EVs from *L. crispatus* BC5 and *L. gasseri* BC12 promote lactobacilli adhesion on cervical HeLa cells and reduce the adhesion of opportunistic pathogens [81].

### 2.2. Mechanisms Relying on Lactobacilli Interactions

Besides releasing biological molecules or structures modulating microbial adhesion, lactobacilli cells themselves can interfere with such a process: by displaying a variety of surface structures, the so-called adhesins, lactobacilli mediate the interaction with the host epithelium and other microorganisms. Adhesins are mainly multi-functional cytoplasmatic proteins, exerting moonlighting functions when expressed on the cell surface; some are glycosylated [82]. Viable lactobacilli are able to co-aggregate with bacterial or viral particles, thus masking pathogen surface molecules that mediate the recognition of specific receptors on the host epithelium. As an example, *Lactobacillus delbrueckii* strain 45E, isolated from a woman’s anogenital region, counteracts genitourinary infections by co-aggregating with Group B *Streptococcus* and, to a minor extent, with *E. coli* and *Klebsiella* spp., possibly through the interaction of cell-surface proteins, such as mucin-, fibronectin-, and collagen-binding proteins displayed by the *Lactobacillus* strain [83]. Analogously, vaginal *L. crispatus* strains, namely BC1 and BC3, show fast and high levels of co-aggregation with piliated *N. gonorrhoeae*, although the molecular determinants have not been identified [78]. *Lacticaseibacillus rhamnosus* GG co-aggregation with *S. aureus* is mediated by the presence of pili since pilin subunits SpaA, SpaB and SpaC mutation in *L. rhamnosus* GG significantly decreases co-aggregation [84].

Lactobacilli can also play a competitive exclusion of pathogens, thus impairing their attachment to the host epithelium. It has been proposed that many *Lactobacillus* strains display a higher affinity for epithelial surface receptors than pathogenic microorganisms. In this regard, various *Lactobacillus acidophilus*, *L. gasseri* and *L. jensenii* isolates from healthy premenopausal vaginas effectively antagonize the adhesion of urogenital pathogens such as *G. vaginalis* to the vaginal mucosa. *L. acidophilus* and *L. gasseri* competitive activity seems to be mediated by glycoproteins, while carbohydrates are mainly involved in *L. jensenii* adherence; vaginal cells express surface glycolipids, which presumably are the targets of the competition observed between the lactobacilli and the pathogenic microbes [85]. It has also been demonstrated that vaginal isolates of lactobacilli are able to interfere with *C. albicans* adhesion to cervical cells both by preventing yeast adhesion or competing with the pathogen. Such effect is not strictly dependent on the formation of a “shell” of *Lactobacillus* on the surface of epithelial cells; rather, it should be mediated by specific ligand-receptor interactions. Indeed, a very high level of *Lactobacillus* adhesion on cervical cells is not required to observe a protective effect [86].

Another model of competition involving resident vaginal lactobacilli and pathogenic species is based on the availability of nutrients: both kinds of microorganisms preferentially utilize simple forms of carbohydrates and require essential elements, consuming the nutrients present in the vaginal niche. In this regard, it has been reported that glucose depletion represents an additional mechanism of action for lactobacilli antagonism towards *C. trachomatis* since, in a panel of vaginal lactobacilli, the strains of *L. crispatus* displaying the highest consumption of glucose are also the most effective in reducing *C. trachomatis* cellular infection. In addition, in lactic acid bacteria, glucose fermentation is connected to the production of organic acids with antimicrobial activity, including lactic acid, therefore fostering the defensive strategies of vaginal beneficial lactobacilli [39].

Additionally, lactobacilli and other members of the microbiota perform an immunomodulatory action in the human host by modulating the homeostasis of the immune system, thus assuring a healthy and functional vaginal tract. Vaginal lactobacilli induce the production of chemokines and cytokines by epithelial cells. In particular, *L. crispatus* isolates induce, in HeLa and J774 cells, the expression of the anti-inflammatory cytokine IL-10 while inhibiting the expression of other pro-inflammatory cytokines such as IL-6, IL-8 and TNF-α [87]. Further studies have shown that the presence of particular CSTs affects the levels of cytokines and chemokines produced by the host’s epithelial cells: women falling in CST IV (dominance of anaerobic or strict anaerobic organisms) show higher levels of IL-1α, IL-1β, TNF-α, IFN-γ, IL-4, IL-8, IL-10 and IL-12p70, compared to CST I women, being CST IV characterized by the greatest pro-inflammatory activity [88].

Noteworthy, the vaginal ecosystem includes cells of innate and adaptive immunity, in addition to epithelial cells and microbiota. Macrophages, neutrophils, dendritic cells, Langerhans cells, NK cells and T and B lymphocytes are the most abundant. Activation of immune cells in the female reproductive tract is strictly mediated by the expression of Pattern Recognition Receptors (PRRs), primarily regulated by endocrine signaling of sex hormones, including estrogen [89]. PRRs recognize conserved molecular structures known as microorganism-associated molecular patterns (MAMP) and induce the production of cytokines, chemokines, and other innate effectors. Stimulation of PRRs by microbial cells activates cytokine and chemokine signaling cascades, resulting in the production of IL-1β, IL-6, IL-8 and TNF-α which, in turn, activate various immune cells such as NK cells, macrophages, T-cytotoxic and T-helper lymphocytes and B lymphocytes [90]. Among PRRs, the best-known and most studied signaling mechanism involves Toll-like receptors (TLR), which are expressed both on the squamous epithelial cells covering the vagina and on the columnar epithelial cells that protect the upper part of the female genital system. The TLR group comprises 10 members. TLRs 1, 2, 4, 5, 6 and 11 mainly recognize microbial membrane components and are expressed on the cell surface, while TLRs 3, 7, 8 and 9 detect nucleic acids of bacterial and viral origin and are expressed in the endoplasmic reticulum, endosomes, lysosomes and endolysosomes. Colonization of in vitro cellular multilayers by common vaginal commensals, including *L. crispatus* and *L. jensenii*, attenuate pro-inflammatory outcomes, reducing IL-6, IL-8 and TNF-α secretion after TLR stimulation, demonstrating their active role in modulating inflammation [91]. In particular, vaginal *L. crispatus* and *L. jensenii* strains prevent pro-inflammatory activity by decreasing the levels of pro-inflammatory cytokines IL-1α and IL-8. This immunomodulatory action is fundamental in women suffering from bacterial vaginosis, in which the concentration of IL-1β cytokine is high, since *G. vaginalis* stimulates the increase in the production of pro-inflammatory cytokines (IL-1β, IL-8 and IL-6) and antimicrobial substances such as defensins, producing an inflammatory response [88].

## 3. Sexually Transmitted Infections (STIs) and Viral STIs

### 3.1. STIs Public Health Issues

STIs represent a significant public health concern worldwide with a direct impact on sexual and reproductive well-being, including infertility, pregnancy complications, cancer and increased risk of human immunodeficiency virus (HIV) infection [92,93]. Most STIs show no symptoms in women and require screening for timely diagnosis and treatment; however, if untreated, they can lead to dramatic consequences and heavily affect the quality of life. Since STIs involve some sensitive and strictly private aspects of life or are prevalent in vulnerable or marginalized groups of people, they are also associated with shaming, blaming, stigmatization and violence, with the result of ignored or hidden epidemics [94]. Actually, STIs are among the most common infections affecting humans and cause significant morbidity and mortality globally, resulting in 2.5 million deaths and 1.2 million cases of cancer per year [95,96].

STIs are caused by a broad range of bacteria, fungi, viruses, and parasites that are transmitted through unprotected sexual contact, including vaginal, anal, and oral sex. Co-infections with multiple pathogens are very frequent among people with STIs and can result in enhanced infectivity and more severe clinical manifestations [97,98,99,100,101,102]. Sexually transmitted co-infections are prevalent due to the shared transmission routes and asymptomatic infections that result in untreated cases. Previously acquired STIs could favor new infections by impairing the mucosal barrier integrity, cellular homeostasis, microbial eubiosis and the inflammatory microenvironment [98,103,104]. To promote early and fast diagnosis, multiplex rapid detection tests are available [105,106].

### 3.2. STIs Transmission

High-risk behaviors, such as inconsistent condom use and multiple sexual partners, significantly increase the risk of co-infection with multiple pathogens [107]. In addition, low income, poor education, and young age conditions contribute to this elevated risk [108,109]. Although sexual contact is the primary mode of transmission, some pathogens responsible for STIs can also be transmitted from mother to child during pregnancy, childbirth, breastfeeding, or through blood products and tissue transfer [96,110,111]. STIs can be acquired at any age, but recent epidemiological data highlight a growing concern among adolescents and young adults (aged 15–24 years), especially from lower socioeconomic backgrounds [112] and ethnic minorities [113]. Adolescents represent about one-fourth of the sexually active population and account for half of new STIs every year, and 10 million only in the United States [95,114,115].

The four most common curable STIs—syphilis (*Treponema pallidum*), gonorrhea (*N. gonorrhoeae*), chlamydia (*C. trachomatis*) and trichomoniasis (*T. vaginalis*)—cause more than one million infections each day, with steadily increasing numbers. Yet, they are all preventable, easily diagnosed, and treatable with antibiotics, although rapidly increasing multi-resistant gonorrhea is emerging in different regions worldwide [111].

### 3.3. Viral STIs Epidemiology and Public Health Prevention Strategies

In this review, we focus on viral STIs caused by human papillomavirus (HPV), human immunodeficiency virus (HIV), and genital herpes simplex virus (HSV). These infections can cause devastating diseases such as cancer, acquired immunodeficiency syndrome (AIDS) or different severe complications in adults and neonates. Treatment options are limited or missing; for example, vaccines are available for HPV to prevent infection that can lead to cervical cancer, but only against a few of the hundreds of HPV genotypes [116,117]. HIV and HSV establish infections for life, with lifelong latency phases in non-permissive “reservoir” cells. Many antiviral treatments, such as acyclovir [118] and ART [119,120], can suppress viral replication, but currently, there is no vaccine for HSV [121,122] nor HIV [123,124,125], nor definitive cures exist for any of these viral STIs, which remain cause for utmost concern.

Viral STIs are alarmingly widespread worldwide but with dramatic geographical, social, age and gender inequalities. According to the WHO’s most recent data [96,126], in 2022, about 300 million women were living with HPV infection, the primary cause of cervical cancer, and 662,000 new cases of cervical cancer and 349,000 cervical cancer deaths were estimated globally [96]. Despite the global increasing coverage of HPV vaccination, cervical cancer remains the most common cancer in women in 25 countries, many of which are in sub-Saharan Africa [127]. People living with HIV reached 39 million by 2022 [96], two-thirds of whom are in the African region. More than half of the new HIV infections occur among individuals belonging to minority groups and their partners. Among adolescents and young people aged 15–24 years, a disproportionate number of new cases of HIV infection are still occurring in females in sub-Saharan Africa. In 2022, HIV globally claimed 630,000 lives, 13% of these occurring in children under the age of 15 years [128]. Genital infections by HSV affect more than 500 million people aged 15–49 years [129]. Although HSV-2 typically causes genital herpes and HSV-1 typically causes orolabial herpes, the prevalence of genital herpes by HSV-1 has increased significantly in the last twenty years, mainly in adolescents [130,131,132,133,134,135]. A plausible explanation is that the decreased acquisition of HSV-1 in childhood makes two-thirds of people reach sexual debut without prior exposure to the virus and immunologically naïve, entailing an increased risk of genital acquisition in adulthood [135,136]. Increasingly common oral sex practices among adolescents may also contribute to this HSV-1 epidemiologic change [137,138]. Considering that in the world, 3.7 billion people under age 50 (67%) have HSV-1 infection [129,139], this emerging shift in the HSV-1 pattern of spread foreshadows worrisome scenarios, prompting WHO and global partners to lead an international, multidisciplinary effort to urgently develop an HSV vaccine [121]. The COVID-19 pandemic and disruption in public health services have had a significant impact on STI-related prevention and care activities, causing a reduction in STI screening, diagnosis and treatment, ultimately increasing the number of infected asymptomatic people unaware of their status [140,141].

The recent outbreaks caused by emerging or re-emerging viruses, such as monkeypox, Ebola, and Zika viruses, which can be acquired by sexual contact present a growing challenge to the provision of adequate services for the prevention and control of viral STIs. The sudden recent monkeypox global outbreak of May 2022 [142,143], with its unusual epidemiological characteristics, has involved so far 117 states, with more than 97,000 confirmed cases and 186 deaths [144,145], and currently, more WHO public health emergency alerts have been issued. To face future challenges, the WHO has designated STI prevention as a key priority for 2022–2030 and has defined the strategies of the global health sector to pursue the ambitious targets of eliminating AIDS, viral hepatitis B and C, and STIs by 2030 as public health concerns [96]. Despite ongoing progress, the latest data show that many indicators remain off-track to achieve the 2025 and 2030 global targets [95,146]. The coverage of HPV vaccination among girls by age 15 increased from 14% in 2020 to 17% in 2022 worldwide, but there is still a long way to go to reach the global coverage target of 50% by 2025 and 90% by 2030, considered a crucial step in achieving cervical cancer elimination [95,96,126]. Also, HIV treatment has reached unprecedented levels and results, with over 75% of people living with HIV globally receiving ART therapy by the end of 2022. However, 12.6 million people in the world are still living with untreated or unsuppressed HIV [128]. In addition, progress is dramatically unequal by country, sex and population groups, and HIV-related deaths remain unacceptably high. While sub-Saharan Africa has high treatment coverage due to effective efforts, less than 50% of people living with HIV in 26 low- and middle-income countries were receiving antiretroviral therapy in 2022 [95,96]. This highlights the necessity for intensified efforts across all disease areas [146].

## 4. Lactobacilli and Viral STIs

Viral STIs remain a significant public health concern worldwide due to their prevalence and associated complications. Therefore, there is a growing need for alternative prophylactic and therapeutic approaches accessible to a wider range of patients. The development of probiotic lactobacilli, or their postbiotic derivatives, as a new strategy for treating various vaginal infections has progressed rapidly [23,80,81,147,148,149]. Vaginal lactobacilli play a crucial role in maintaining vaginal health by sustaining an acidic environment, producing antimicrobial compounds, and modulating immune responses. A large amount of evidence proved that these beneficial bacteria may confer protection against viral STIs [150,151,152]. Increasingly more studies highlighted the associations between a *Lactobacillus*-dominated vaginal microbiota and reduced viral STI prevalence [9,153,154,155,156,157,158]; however, the biological and molecular mechanisms involved remain poorly defined. In this review, we collected recent evidence regarding the anti-viral activity of vaginal lactobacilli and the proposed molecular mechanisms, which are summarized in Table 1.

Understanding the mechanisms by which vaginal lactobacilli exert protective effects against sexually transmitted viruses is of significant interest for several reasons: (i) it could offer insights into novel preventive strategies to reduce viral STIs transmission rates and associated morbidity, with remarkable public health implications; (ii) it can provide valuable information into the dynamics of the vaginal microbiota and its impact on viral STIs acquisition and transmission, elucidating the role of singular specific microbiota components; (iii) it can promote the development and optimization of probiotic formulations tailored to enhance vaginal health and reduce susceptibility to viral STIs; (iv) it can shed light on the complex interplay between host immunity, microbial communities, and viral pathogens within the vaginal microenvironment, potentially leading to the identification of new targets for intervention.

### 4.1. HPV

HPV is a small double-stranded DNA virus and the most common sexually transmitted virus, highly prevalent among women of reproductive age. It is the first cause of cervical cancer in women and anogenital tumors in men who have sex with men, but it can also be associated with cancers of the head and neck, as well as with anogenital warts and respiratory papillomatosis [176].

To date, more than 200 genotypes have been characterized and classified as carcinogenic high-risk (HR) or low-risk (LR) HPV. Although the primary infection is asymptomatic and usually spontaneously resolves in 90% of cases within 12–24 months, persistent infection with HR-HPV, mainly genotypes 16 and 18, may lead to cervical intraepithelial neoplasia (CIN), which, if untreated, may progress to squamous intraepithelial lesions (SIL), classified into low-grade (LSIL), high-grade (HSIL) and cell carcinoma (SCC). HPV16 and HPV18, together, are responsible for almost 70% of cases of cervical cancer worldwide [177].

HPV targets the basal layer of the multi-stratified squamous epithelium, penetrating through abrasions or epithelial barrier disruption of the cervicovaginal mucosa. The productive HPV replication cycle deeply correlates with the differentiation state of infected cells. The episomal viral DNA genome spreads to proliferating basal layer daughter cells during mitosis, providing a permanent pool of infected cells, while HPV proteins are differentially expressed as the virus migrates towards the differentiated and resting spinous layer cells, where virions assembly is completed, and viral progeny is released [178].

Although uncommon, active HPV infection can last even decades thanks to several immune evasion mechanisms implemented by the virus, which induce a downregulation of the expression of interferon, Toll-like receptor 9 (TLR 9) and major histocompatibility complex class I (MHC-I), as well as chemotactic and pro-inflammatory factors [179].

Long-lasting HPV persistent infection favors viral circular DNA genome integration into the cellular genome, promoting the switch from productive infection towards neoplastic transformation. The linearization of integrated viral DNA leads to the disruption of the HPV E2 gene and to the consequent up-regulation of HPV E6 and E7 oncoproteins, the main key drivers of malignant transition and progression, by the impairment of onco-suppressor gene (pRB and p53) control [178,180,181,182,183]. Moreover, viral integration into the host genome can trigger genome instability and possibly upregulation of tumor-related genes and accumulation of additional mutations [184,185]. Cervical cancer is one of the few cancers that can be prevented through both vaccination (bi, -tetra- and nonavalent recombinant L capsid subunit-based vaccines) and screening (Pap test and HPV DNA detection) [186]. As current vaccines do not cover all high-risk HPV types, high-quality screening and education programs are essential to prevent cervical cancer, especially in developing countries.

A few studies have reported a clear association between cervicovaginal microbiota composition and HPV prevalence or infection outcome in HR-HPV-positive women [157,187,188,189], although their causal relationship remains unclear [89,190,191,192]. The relationship between cervicovaginal microbiota and HPV infection and cervical cancer was recently analyzed in Latina women since they are extremely susceptible to HPV, with an incidence exceeding 40% and a chance of developing cervical cancer 40% higher compared to other racial-ethnic groups [193]. A healthy microbiome in Latinas was associated with the enrichment of *L. crispatus*, *L. iners*, *Anaeroccoccus*, and *Coriobacteriaceae*. In conditions related to abnormal cytology, *L. iners* and *L. crispatus* were depleted, while *Sneathia* spp., *C. trachomatis* and *G. vaginalis* became prevalent. The cervicovaginal microbiota of Latinas with cervical cancer exhibited an increased abundance of *Fusobacterium* and *Sneathia* spp. and depletion of *L. crispatus*, confirming the protective role of lactobacilli against HPV infection, neoplastic progression and cancer development. In a recent longitudinal study, Molina et al. [157] investigated by high-resolution RNA sequencing technology the temporal cervicovaginal microbiome profile in 141 HR-HPV DNA-positive women with normal cytology at first visit, of whom 51 were diagnosed by cytology with SIL six months later. They found that women with high diversity and *Lactobacillus* depleted microbial community at first visit had a higher risk of developing SIL, suggesting that the detailed cervicovaginal microbiome composition could be used as a biomarker for early detection and treatment of SIL after HR-HPV infection diagnosis. Although it is well established that BV favors HPV infection, persistence and neoplastic progression [194,195,196,197], the reverse association has also been reported. HPV infection can alter the vaginal microbiome by down-regulating the host mucosal innate peptides (defensins), elafin and S100A7, used by lactobacilli as amino acid sources, highlighting a causal relationship between HPV infection and BV onset [198]. Indeed, E7 oncoprotein dramatically downregulates defensin expression. The reduced availability of defense peptides, working as nutrients for lactobacilli, results, therefore, in a negative impact on *Lactobacillus* species survival and in an imbalanced vaginal flora. BV establishment and persistence would then lead to oxidative stress that promotes the progression of HPV-related pre-neoplastic lesions. Taken together, this body of evidence indicates that the association between HPV and BV is articulate and bidirectional. Moreover, HPV infection impacts significant microbial changes in the microbial community of different body sites (cervix, vagina, urethra and rectum). Intervening in the microbiome of non-diseased areas might help establish a stable, healthy microbiome to prevent and manage diseases [159].

Vaginal lactobacilli exert their protective role against HPV infection, persistence and progression towards neoplastic and cancer development through multiple mechanisms. For instance, they can contribute to strengthening the mucosal epithelial barrier, avoiding pathogen entry, mitigating or inhibiting chronic inflammation conditions, modulating host clearance ability, or impairing molecular pathways involved in carcinogenesis [104,192,199].

A possible effect of vaginal pH value on HPV infection has been investigated in in vivo and in vitro models: acidic pH values determined by lactobacilli prevalence in eubiotic conditions would impair the adhesion capability of HPV to host cells by reducing the pseudovirions binding to syndecan-1 receptor [160].

In order to investigate the protective mechanisms of lactobacilli against HPV infection and cervical cancer development, Pawar et al. [161] analyzed the effect of cell-free culture supernatants (CFCs) of twelve *Lactobacillus* species on HPV18+ (HeLa) and HPV16+ (SiHa) cancer cell lines. They observed that *L. vaginalis* and *L. salivarius* CFCs show the highest antiproliferative activity on HeLa and SiLa cell lines, as measured by MTT assay, probably due to their ability to produce L-lactic acid and H_2_O_2_. In addition, both cell lines treated with lactobacilli CFCs were found to have significantly increased E-cadherin amounts and decreased matrix metalloproteinase 9 (MMP9) levels. Noteworthy, E-cadherin abundance is considered a positive biomarker of the health and integrity status of the epithelial mucosal barrier, promoting the functional architecture of tight junctions that could prevent HPV infection. Under various disease conditions, E-cadherin can be proteolytically cleaved, releasing a soluble E-cadherin fragment and triggering the progressive disassembly of cell junctions [200,201]. Notably, also in a mouse model, *L. crispatus* CCFM1339 led to a significant decrease in the secretion of E-cadherin and a rise in the anti-inflammatory cytokine IL-10, ultimately relieving vaginal inflammation [162]. MMPs are, instead, usually implicated in the breakdown of the extracellular matrix and deeply involved in the invasivity of cancer cells and metastasis [202] and their levels are upregulated in several carcinomas [203]. The increase of E-cadherin and decrease of MMP9 levels observed in this cell culture model suggest a possible role of lactobacilli supernatants as antiproliferative and antimetastatic agents in vivo. Similarly, Liu et al. found a negative correlation between *Lactobacillus* 16S rRNA expression and epithelial-mesenchymal transition-related factors driving metastasis initiation in cancer progression, such as E-cadherin, β-catenin, N-cadherin, and vimentin in postmenopausal SILs and SCC [204].

Moreover, vaginal lactobacilli play an essential role in shaping the immune response accounting for HPV clearance [191]. A recent study has shown that in ectocervical Ect1/E6E7 cells treated with poly (I:C) immunostimulant, *L. gasseri* LGV03 significantly upregulated IFN-α and IFN-β mRNA expression by NF-kB pathway activation and decreased pro-inflammatory cytokines (IL-6 and IL-1β) and chemokine (IL-8) levels [163]. This immune balance would enhance the antiviral activity of the innate immune system and reduce inflammation damage during persistent infection. Butyrate produced by *L. gasseri* LGV03 could also enhance HPV clearance via the restoration of host immunity in an IRF3-dependent manner [205].

To elucidate how a dysbiotic environment could promote HPV infection and persistence, Nicolò et al. [150] examined the effect of individual vaginal *Lactobacillus* species and dysbiosis-associated bacteria on cervical epithelial cells viability, immune homeostasis and antiviral defenses. They observed that supernatants or lysates from different lactobacilli maintained or enhanced HPV16+ SiHa cells viability and metabolic activity and induced optimal levels of IFN-γ and low levels of IL-17 in human mononuclear cells from peripheral blood (PBMCs), then ensuring a balanced cervical environment. In contrast, vaginal dysbiosis-associated bacteria, especially *A. vaginae* (now *F. vaginae*) and partially also *L. iners*, impaired cell viability and epithelial integrity and induce the production of IL-17 and pro-inflammatory cytokines. IL-17 modulates the immune response, inducing an environment hyperinflammatory status and weakening immune antiviral defense, thus exacerbating HPV disease severity and promoting tumor progression [206,207,208]. These findings confirm the crucial role of a healthy vaginal microbiome in protecting against infections and maintaining cervicovaginal health and underscores the importance of microbial balance for effective immune and antiviral responses in the lower genital tract. The same research group has recently identified a possible mechanism by which vaginal lactobacilli protect against the oncogenic progression of HPV-infected cells [164]. They reported that in an in vitro model, different vaginal bacteria species affect the expression levels of HPV-E6 and E7 oncogenes, along with that of p53 and pRB onco-suppressor proteins. Specifically, in HPV16-transformed SiHa cells, *L. crispatus* and *L. iners* significantly decreased E6 gene expression, while *G. vaginalis*, *Megasphaera micronuciformis* and *F. vaginae* increased it [164]. The production of the E7 protein was significantly impaired in cells co-cultured with *L. crispatus* and *L. gasseri* but considerably enhanced co-culturing with *L. iners*, *G. vaginalis*, and *M. micronuciformis*. p53 and pRb amounts were significantly reduced in SiHa cultures exposed to *M. micronuciformis*, and more cells were detected in S-phase with respect to untreated control cells. These data confirm the protective role of lactobacilli against neoplastic initiation, while other species, such as *M. micronuciformis* and, to a lesser extent, *G. vaginalis*, could contribute to cell cycle deregulation. Other non-vaginal *Lactobacillus* strains hold promise as anti-cervical cancer probiotics. For instance, *Lacticaseibacillus casei* LH23 inhibits HeLa cancer cell proliferation, slows down cell migration, reduces histone H3K9 acetylation and suppresses HPV E6 and E7 expression [209,210]. Similarly, the administration of *L. casei* and *L. paracasei* strains to HeLa cells enhances the expression of apoptotic genes like BAX, BAD, caspase-8, caspase-3, and caspase-9, downregulating the anti-apoptotic Bcl-2 gene, performing a general anticancer activity [211].

### 4.2. HIV

HIV is a single-stranded RNA Retrovirus, which mainly targets CD4+ T cells, inducing progressive and severe immune dysfunction leading to AIDS. The widespread implementation of antiretroviral therapy and pre-exposure prophylaxis (PrEP) have significantly reduced HIV transmission; however, HIV and AIDS persist as a significant global health concern, especially in sub-Saharan Africa, where 58% of new infections are among women, including young women and adolescent girls [212]. The extraordinary susceptibility of these women to HIV depends partly on social and behavioral factors but more often on intrinsic host factors [213]. A substantial body of literature has shown a clear association between the elevated risk of HIV in African, Caribbean and Black (ACB) women and the far higher prevalence of BV in these communities, wherein the vaginal microbiota is predominated by different pro-inflammatory anaerobic bacteria. Conversely, in BV-free conditions, *L. iners* results prevalent [18,153,154]. A longitudinal prospective study reported that in South Africa, young women colonized with a highly diverse bacterial community (CST IV) had a 4.4-fold increased risk of acquiring HIV compared with women with *L. crispatus*-dominant microbiota. The *G. vaginalis*-dominated (CST III) cervicotype showed a tendency to high risk of HIV acquisition. Noteworthy, no woman with *L. crispatus*-dominated vaginal microbiota acquired HIV [154]. Another nested case–control study in African women confirmed that vaginal bacterial diversity and several BV-associated bacterial species were significantly associated with a higher risk of HIV acquisition [214]. Further studies investigated the cellular, immunological or molecular mechanisms of anti-HIV protection previously observed at the epidemiological level. Most HIV acquisition in women occurs through unprotected sexual intercourses. Before reaching the CD4+ target cells, the virus must overcome the multiple immune defenses of the genital mucosa, such as virion trapping by mucus [215], degradation by innate antimicrobial peptides [216] and epithelial barrier crossing [217]. Genital mucosal inflammation induced by BV or other STIs can increase HIV acquisition risk by suppressing these innate defense mechanisms; indeed, pro-inflammatory cytokines, e.g., IL-1α, can damage the mucosal barrier, thus facilitating HIV penetration [218,219,220]. In addition, inflammation and chemokines like IP-10 and MIP-1β trigger the recruitment to genital mucosa of activated CD4+T cells, susceptible to HIV, promoting HIV spread [218,221,222]. BV or dysbiotic bacteria can also directly contribute to HIV penetration, suppressing wound healing and affecting the mucosal barrier integrity through sialidase production that degrades cervical mucus [215].

Instead, lactobacilli exert their protective effect against HIV infection both indirectly, by promoting the exclusion of bacteria associated with suboptimal vaginal flora and inducing pro-inflammatory responses, and directly, through the production of metabolites like lactic acid, H_2_O_2_, and short-chain fatty acids (SCFA) that alter the functional structure of the virus or inhibit host susceptibility to the virus in different ways [221]. Concerning H_2_O_2_, an early in vitro study on HIV-1 demonstrated a direct virucidal effect on HIV virions [53]. However, the significance of this result is controversial, particularly in relation to the in vivo physiological hypoxic condition of cervicovaginal mucus. It cannot be ruled out that the locally produced H_2_O_2_ by lactobacilli may have an immunomodulatory effect on the vaginal mucosa, affecting susceptibility to HIV [48].

Lactic acid has a pleiotropic antiviral effect. The direct antiviral activity of lactic acid on HIV virions has been confirmed in many in vitro and ex vivo studies [165,166,167]. First of all, fluorescent HIV-1 pseudoviral particle mobility in eubiotic and dysbiotic cervicovaginal mucus was tracked using high-resolution microscopy: HIV-1 was trapped in cervicovaginal mucus containing relatively high concentrations of D-lactic acid and an *L. crispatus*-dominant microbiota [223]. On the other hand, mobility was not affected at low D-lactic acid concentration, as well as in the presence of *L. iners* or dysbiotic bacteria. In ex vivo experiments using native undiluted cervicovaginal mucus, the strongest activity against HIV was exerted by the protonated uncharged form of lactic acid produced by *L. crispatus*-dominated microbiota; the charged, anion form was inactive [167]. In addition, lactic acid produced by vaginal lactobacilli plays an important role in enhancing the integrity of the cervicovaginal epithelial barrier to protect against infection with HIV and other STIs. Metaproteomics and transcriptional analyses in human ectocervical and vaginal cell lines demonstrated that culture supernatants from *Lactobacillus* spp. producing high levels of lactic acid, or treatment with protonated lactic acid, alter the expression of genes related to epithelial barrier integrity and upregulate the expression of tight junction proteins, such as claudin-1, claudin-4 and tight junction protein-2 (TJP-2) [168]. Treatment of vaginal epithelial cells with lactic acid and SCFAs from eubiotic vaginal lactobacilli, including butyric, succinic and acetic acids, can positively modulate the levels of epithelial barrier integrity markers and the expression of cell–cell adhesion proteins [169]. In addition, inflammatory mediators, such as TNF-α, RANTES, IL-6 and IL-8, are reduced by lactic acid and SCFAs of eubiotic lactobacilli, while dysbiotic bacteria increase them [41,169]. These results indicate that protonated lactic acid, predominating at pH levels below 3.86 eubiotic condition, is a major anti-HIV-1 metabolite present in acidic cervicovaginal fluid. Its protective effect can be dampened by the increased pH typical of a dysbiotic environment, suggesting a key role for lactic acid in future prevention strategies. Lastly, vaginal lactobacilli exert an additional mechanism of protection against HIV infection via the release of extracellular vesicles (EVs). EVs from various strains of *Lactobacillus*, isolated from vaginas of healthy women, inhibit HIV-1 replication in immortalized human T cells in vitro and in human cervicovaginal and tonsillar tissues ex vivo, likely affecting HIV gp120 properties [38,80].

### 4.3. HSV

There is evidence of the role of lactobacilli in protecting against herpesvirus infection; both longitudinal and cross-sectional epidemiological studies indicate the presence of the infection or increased frequency of its acquisition in women experiencing vaginal dysbiosis, entailing vaginitis and especially BV [224]. The herpesviruses that are mainly responsible for STIs are HSV-2 (genital serotype), with a recent incidence increase of HSV-1 (facial serotype). Only a few reports mention the role of human cytomegalovirus (HCMV), too [225]. The directionality of the relationship between healthy or perturbed vaginal microbiota and herpesvirus infection or recurrence in the vaginal tract is still under study: in one direction, the lactobacilli could prevent HSV infection or reduce shedding upon reactivation; in the other direction, the presence of HSV lesions favor inflammation, the alteration of vaginal microbiota and the occurrence of BV. This kind of correlative evidence is widely reported. However, no systematic study or clinical trial has been performed where pharmacological interventions on one or both players (e.g., antibiotic treatment against dysbiotic vaginal microbiota or antiviral treatment against HSV) would highlight the preferential direction of the interaction. The only completed clinical trial [226] was performed on a limited number of HSV-2 positive women (n=12) and aimed to determine the effect on HSV-2 shedding of asymptomatic BV untreated or treated with metronidazole. Nevertheless, the results of the trial were not made publicly available. A recent report [227] analyzed the effects of HSV-2 reactivation and shedding on the development of BV. In a one-way crossover study on 41 participants, the effect of valacyclovir administration was evaluated on the suppression of BV manifestation, checking the vaginal microbiota composition by taxon-specific qPCR and the Nugent score. The results highlighted a clear reduction of viral production after antiviral treatment but no significant change in the composition of the vaginal microbiota, indicating no shift towards the healthy bacterial species (*L. crispatus* and *L. jensenii*) nor towards the neutral (*L. iners*) or dysbiotic species (BV-Associated Bacterium-2 and *Megasphaera*). This study admittedly did not consider the effect of anti-BV treatments on HSV-2 replication and shedding, but, based on previous results in which women with BV showed an increased risk of HSV-2 infection [224,228], a role of the healthy microbiota in protecting from HSV-2 primary infection is expected. These mechanisms have been evaluated at the cellular or molecular level only in a limited number of studies, in particular for HSV-2 and, in a few instances, for HSV-1. Since the two viruses are highly similar in genomic composition and molecular mechanisms for entry and replication, it is conceivable and expected that the findings for one virus will recapitulate or suggest similar outcomes for the other. An ex vivo multilayer culture system of vaginal epithelial cells (VEC) was exploited to assay the effect of different bacterial species on HSV-2 infection and replication [229]. The cultures were colonized by different vaginal microbiome communities resembling the composition of different vaginal states, ranging from the dysbiotic one predominated by *Staphylococcus* spp. to the healthy one dominated by *Lactobacillus* spp. prior infection with HSV-2. HSV-2 titers were lower in the *Lactobacillus*-dominated microbiota as compared to non-colonized ex vivo cultures or cultures colonized with other bacteria, recapitulating the epidemiological studies that indicate an increased risk of seroconversion for HSV-2 for women lacking a healthy vaginal microbiota [224,230]. In a greater close-up, the ability of *L. crispatus* to block HSV-2 infection of simian Vero cells and human HeLa cells was analyzed [170]. Microcolonies of *L. crispatus* formed on the surface of treated cells would hamper HSV-2 entry by preventing the binding of the virus to the viral receptors. This accounts for the direct effect of intact bacterial cells on virus infectivity. These results were in line with those of previous reports, linking the antiviral effect to the ability of specific *Lactobacillus* strains to adhere [151,152,171]. We recently reported the antiviral effect of cell-free supernatants of *L. crispatus* and *L. gasseri* cultures on the replication of HSV-1 [172], highlighting that not only intact cells, but also a cocktail of *Lactobacillus* metabolites may exert an antiviral function.

Taken together, in vitro experiments and epidemiological studies at present suggest the antiviral activities exerted by healthy *Lactobacillus* species, with mechanisms ranging from the maintenance of natural defense barriers to direct virucide activity. Some lactobacilli metabolites are known for their toxicity against HSV-2, e.g., H_2_O_2_ and lactic acid. In particular, the acidic pH determined by lactic acid has the ability to permanently inactivate the fusogenic glycoprotein apparatus of the HSV envelope [173,174,231]. An effect could be determined by isolated components of lactobacilli (e.g., cell wall) on cell permissivity, i.e., the ability to sustain HSV-2 replication [171]. The modified cellular pathways are still to be unraveled at the molecular and transcriptomics level.

It has been shown that HSV-1 is adhesively trapped by the cervical mucus even if the mucus native mesh structure is not tight enough and consists of pores with a bigger size compared to the virus particle [175]. Therefore, mucus provides a mechanism against HSV different from steric blockade, possibly due to the hydrophobic interactions with the viral envelope. This does not apply to small capsid naked, non-enveloped viruses that diffuse in mucus at the same speed as they do through water [232]. These findings underscore the importance of mucus integrity as a protection factor against HSV infection. *Lactobacillus* species of the healthy vaginal microbiota do not produce mucin-degrading enzymes (mucinase, sialidase, glycosulfatase, prolidase, etc.) as the dysbiotic BV bacteria do [233,234,235]; therefore, their dominance in the vaginal milieu contributes to a general anti-herpesvirus activity.

### 4.4. Other Viruses

Apart from the typical viruses that cause localized genital infections described above, more viruses are becoming a focus of interest for the possibility of their sexual transmission, on top of other major routes. Examples come from the positive polarity single-stranded RNA viruses belonging to the Flaviviridae family. One member is hepatitis C virus (HCV), which is transmitted mainly by blood contact and for which sexual transmission in heterosexual couples is extremely low [236,237]. Other Flaviviridae, designated as arboviruses, as they are mainly transmitted by insect vector bites, are Dengue virus (DENV) and Zika virus (ZIKV). For ZIKV, sexual transmission has been proposed due to the presence of virus particles in genital tract body fluids. In particular, the virus replicates in immune-privileged sites of the male genital tract, shielded from the reaction of the immune system, like the testis [238], and persists up to six months after the end of viremia, leading to unexpected sexual transmission [239]. ZIKV sexual transmission occurs more often from male to female [239,240], and sexual transmission accounts for 2% of disease cases. Due to the limited number of documented cases to date, the role of vaginal microbiota and, in particular, of lactobacilli in protecting from this virus has not been investigated. For Dengue virus, the evidence of sexual transmission is even more scarce and anecdotal [241].

On the contrary, the sexual transmission of the Hepatitis B virus (HBV, a DNA reverse-transcribing virus) has been documented, and it is the most frequent route in adults [242]. HBV reaches the male genital tract from the systemic route and can chronically produce infectious viruses there. Thus, HBV viral particles can be present at high concentrations in male seminal fluid and, therefore, be transmitted to the female partner or to the fetus [243].

Similarly, the Ebola virus (EBOV, a negative polarity single-stranded RNA virus belonging to the Filoviridae family) can persist, replicate and produce infectious particles for a long time in the male genital tract and can be detected in seminal fluid after the end of viremia. Outbreak reinitiations by sexual transmission have been documented [244,245].

Recently, since the 2022 outbreaks, human-to-human transmission of the monkeypox virus (MPXV) has gained attention, especially after the spread of the virus in non-endemic countries like the US and Europe. MPXV infection is typically considered to occur through respiratory droplets or close contact with lesions [246], but the possibility of sexual transmission has been taken into consideration, too [247]. Indeed, clinical observations on patients examined for other STIs highlighted MPXV coinfection, indicating that the route of infection could be the same. The frequency is more relevant in men who have sex with men; however, some studies have identified MPXV in women, suggesting a previous underestimation of the viral burden and of the risks for women’s health and pregnancy complications [248]. An animal model of MPXV infection highlighted the susceptibility of the vaginal mucosa, besides the anal mucosa, and hinted that vaginal infection is relevant for subsequent viral shedding [249]. At present, no studies have been published about a possible protective role of lactobacilli against MPXV infection via the vaginal route.

## 5. Towards Lactobacilli as Biotherapeutics for Viral STIs

### 5.1. Drug Resistance in Viral Treatment: A Growing Issue

Currently, antiviral therapy is constantly challenged by the emergence of resistant variants, complicating the effective treatment of viral STIs. For example, chemotherapy is the treatment of choice for cervical cancer caused by high-risk type HPV (i.e., HPV16 and HPV18), but tumor cells can easily become resistant to commonly used chemotherapeutic agents, emphasizing the need for adjuvant therapies to combat the virus at the early stages of infection [250]. Additionally, WHO reported that the prevalence of HIV drug resistance among all individuals receiving treatment ranged from 3% to 29%, posing a serious threat to the prevention of the virus’ spread; moreover, drug resistance can be transmitted to newly infected individuals and resistant strains are likely selected as dominant [251]. Moreover, resistance to HSV therapy, including acyclovir and ganciclovir, is frequently observed in clinical settings due to mutations in the enzymes essential for prodrug activation or in DNA polymerase [252]. For these reasons, there is an urgent need for new therapeutic alternatives, prompting the development of innovative strategies to address the drug resistance phenomenon issue.

### 5.2. Lactobacilli as a Promising Alternative for Treating Viral STIs

Based on the crucial role of lactobacilli in preventing HPV, HIV and HSV infections in vitro and in animal models, further studies have explored the use of lactobacilli-based probiotics as biotherapeutics for viral STIs in women. These studies and trials are summarized in Table 2.

Human trials aimed to investigate the potential of probiotics to enhance genital HPV clearance and improve the quality of cervical smears. In a pilot study, 54 women with HPV infection and LSIL consumed a commercially available probiotic (Yakult) containing *L. casei* Shirota daily for 6 months, and a significant reduction in HPV-associated cytological abnormalities was observed, which was twice as frequent in the intervention group as in the control one. The HPV clearance rates were also higher in the women assuming probiotics, but not significantly, likely due to the small number of subjects involved in the study [253]. A well-designed study was conducted by Ou and colleagues to investigate the influence of the oral probiotic formulation U-relax ^®^, containing *L. rhamnosus* GR-1 and *L. reuteri* RC-14, on 121 women with genital HR-HPV. Although the intake of the probiotic formulation did not influence HPV clearance, after three months of treatment, the rates of low-quality and mildly abnormal cervical smears consistently decreased. This aspect is important because some mild abnormalities do not regress spontaneously but can progress into precancerous lesions or rarely cancer, especially in patients positive for HR-HPV [255]. Similarly, Dellino et al. highlighted that the long-term administration of oral *L. crispatus* M247 in HPV-positive women determined a higher percentage of clearance of PAP-smear abnormalities than in the control group [256]. Since *L. crispatus* M247 is a well-known probiotic strain with proven capacity to colonize the vaginal niche after the passage in the intestine, it has been proposed that the restoration of vaginal balance can strengthen the defenses against HPV, facilitating its clearance. In support of this, another clinical trial reported that 90 days of oral treatment with *L. crispatus* M247 shifted the CST status to CST I (*L. crispatus*-dominated microflora) in most of the HPV-positive women enrolled, alongside an increased HPV clearance rate. At the end of the study, women who still had a CST III or CST IV vaginal microbiota tested positive for HR-HPV or LR-HPV, while only 20% of HPV-positive women had a CST I microbiota [149]. Another piece of evidence supporting the importance of restoring eubiosis in tackling HPV infection has been provided by Palma et al. In this study, *L. rhamnosus* BMX 54 (NORMOGIN ^®^) was topically administered as vaginal tablets for either long (6 months) or short periods (3 months) to 117 women with concurrent HPV and BV or vaginitis who had previously been treated with antimicrobials (metronidazole or fluconazole). Long-term probiotic users not only experienced significantly fewer recurrences of bacterial and vaginal infections compared to those using tablets for only 3 months but also showed improvement in their HPV condition, with increased chances of resolving HPV-related cytological anomalies and an enhanced overall HPV clearance rate [254].

Besides probiotics, another strategy could be the usage of non-viable lactobacilli derivatives, or postbiotics, as biotherapeutics. For instance, lacidophilin is a postbiotic derived from the fermentation of *L. acidophilus* and possesses a broad antibacterial activity. Recent research investigated the combination of lacidophilin vaginal capsules and antitumor rh-IFN-α2b for treating HPV-infected women, with positive outcomes in terms of better microflora restoration and higher negative HPV conversion ratio compared to women receiving only rh-IFN-α2b [257].

In a randomized, double-blind trial, the supplementation of micronutrients combined with *L. rhamnosus* CAN-1 in 21 women assuming therapy for HIV increased CD4+ counts, reduced hospital visits and improved the overall quality of life [260]. The oral intake of other probiotic strains (i.e., *L. rhamnosus* GR-1, GG, and HN001) for at least 30 days has been shown to alleviate diarrhea and delay the decline of CD4+ lymphocytes and morbidity in HIV/AIDS subjects by improving mucosal immune response and enhancing effects of antivirals [266]. In a double-blind placebo-controlled clinical trial, a symbiotic intervention with *L. plantarum* and *Pediococcus acidilactici* was safe and led to a small increase in CD4+/CD8+ ratio in HIV-infected patients [263], whereas the administration of another strain, *L. casei* Shirota, did not provide beneficial effects in HIV-infected patients over a 12-week period [262].

Interestingly, low diversity in vaginal microbiota, primarily dominated by *L. crispatus*, correlates with a reduced risk of HIV acquisition. Instead, high-diversity microflora associated with BV facilitates HIV shedding in women by disrupting the genital epithelial barrier and recruiting highly HIV-susceptible target cells (e.g., activated CD4+ T cells) to the genital mucosa [267]. It also appears that women with a microbiota not dominated by lactobacilli (CST IV) are poorly susceptible to therapy with the antiviral tenofovir, likely due to the capacity of *G. vaginalis* and other BV-associated anaerobic bacteria to quickly metabolize the drug, thus reducing its availability; conversely, in *Lactobacillus*-dominant women, HIV incidence is effectively reduced by 61% after topical application of tenofovir-based gel [268]. These findings suggest that interventions to modulate vaginal microbiota represent an intriguing strategy to reduce incidence rates and improve HIV treatment. In this view, Lactin-V is a promising live biotherapeutic agent containing *L. crispatus* CTV-05, administered vaginally by a pre-filled applicator. Encouragingly, a phase 2 trial found that participants receiving Lactin-V after standard antibiotic treatment for BV had lower recurrence rates than those receiving a placebo, and the strain was still detected in 79% of women for at least 12 weeks after the end of treatment [23]. More recently, it has been shown that the application of Lactin-V in women treated for BV reduced genital inflammation (i.e., IL1α) and biomarkers of epithelial distress (i.e., soluble E-cadherin), opening up the possibility of employing Lactin-V to reduce HIV susceptibility [269].

Acyclovir is the most common first-line therapy for the management of HSV-2, but it is ineffective in eradicating the virus permanently, as it evades the immune system, establishing a lifelong latency in neuronal cells, and in preventing virus transmission [270]. Hence, in seeking alternatives to counteract HSV-2 infection, the use of probiotics could have clinical relevance due to the capacity of some probiotic strains to restore vaginal microbiota and, importantly, to modulate the immune system. For instance, it has been demonstrated that *L. rhamnosus* GG protects mice from HSV-2 by inducing the production of antiviral IFN-I [271], but unfortunately, no data are available for humans. Instead, Mohseni and colleagues conducted a randomized double-blind controlled trial enrolling 81 women suffering from HSV-2 to compare the efficacy and safety of oral acyclovir tablets (400 mg every 12 h for 6 months) and a multi-strain vaginal capsule containing *Levilactobacillus brevis* CD2, KB290 and SBC8803 (twice daily for 6 months). Notably, both treatments produced similar effects in reducing the healing time of the lesions, the overall length of an episode, the time interval between two recurrences, and the duration of viral shedding. The probiotic application was also effective in limiting the severity of pain as antiviral therapy, but without side effects ascribed to acyclovir (headache, nausea, diarrhea and abdominal pain) [265]. In another clinical study, the food supplement Florium containing four lactobacilli strains (*L. crispatus* LBV88, *L. rhamnosus* LBV96, *L. gasseri* LBC150 and *L. jensenii* LBV116) was administered to 30 pregnant women with HSV infection and led to a general improvement of vaginal landscape, as the number of total lactobacilli increased while the pathogens decreased. In addition, symptoms (vaginal discharges, itching, swelling and mucosa redness), as well as complications related to pregnancy (placental insufficiency, preeclampsia and fetal distress incidence), were reduced by approximately two times [264]. These findings, coupled with the easy availability of probiotics and their low cost compared to antiviral therapy, make this approach particularly promising.

## 6. Conclusions

The crucial role of lactobacilli in maintaining vaginal homeostasis is well-established, particularly in preventing and combating bacterial and fungal infections. Evidence suggests that a *Lactobacillus*-rich vaginal microbiota offers protection against sexually transmitted viral infections as well, especially HPV, HIV and HSV.

To date, several studies have been conducted in vitro and in animal models to elucidate molecular mechanisms by which lactobacilli exert activity against viruses, including immunomodulation, production of virucidal metabolites, blocking viral attachment to cells, and reducing permissivity.

In addition, clinical studies have been performed on human subjects with the purpose of evaluating the effectiveness of lactobacilli-based probiotics against viral STIs. Even though the available results are promising and encouraging, the feasibility of translating probiotics into effective biotherapeutics for viral STIs remains controversial. This is due to inconsistencies and, sometimes, inconclusive results, especially regarding HIV-1 infection. Further clinical studies, considering the co-administration of probiotics and drugs, the duration of probiotic treatment, sample size and route of administration, are needed to demonstrate the validity of lactobacilli-based probiotics as anti-HPV, anti-HIV and anti-HSV biotherapeutics.

To date, results collected in this review hold promise for advancing our understanding of vaginal health, viral pathogenesis, and potential preventive and therapeutic strategies based on lactobacilli.

## Figures and Tables

**Table 1:** Mechanisms of protection against viral STIs exerted by lactobacilli.

Virus	*Lactobacillus* Species/Strain	Antiviral Effect	Metabolites and/or Molecular Mechanism	Targeted Organ/Cell Type/Model Cell Line	Ref
HPV	n.d. (combination of species)	reduced virus colonization	cytokine network	cervix, vagina, urethra and rectum	[159]
	n.a.	block of binding to syndecan-1 receptor	acidic pH	293 FT, HeLa, SiHa, and HaCaT	[160]
	*L. vaginalis* *L. salivarius* *L. crispatus* CCFM1339	block of cell proliferation; maintenance of epithelial mucosal barrier integrity	L-lactic acid and H_2_O_2_; E-cadherin increase; MMP-9 decrease	HeLa and SiHa; mouse models	[161,162]
	*L. gasseri* LGV03	enhance innate immune system; reduce inflammation damage	IFN-α and IFN-β upregulation; IL-6, IL1β and IL-8 downregulation	Ect1/E6E7	[163]
	*L. gasseri* *L. jensenii*	cell viability; balanced cervical environment	optimal IFN-γ; low IL-17	SiHa	[150]
	*L. crispatus* *L. iners*	block of oncogenic progression	decrease of E6 and E7 expression	SiHa	[164]
HIV	*L. crispatus* *L. jensenii* *L. acidophilus*	direct virucidal effect; immunomodulation; block of cell susceptibility	H_2_O_2_	CEM, mucosa	[48,53]
	*L. crispatus*	virion trapping	lactic acid (protonated uncharged)	cervicovaginal mucus	[165,166,167]
	n.d. (metabolite mixture mimicking eubiotic conditions)	reduction of viral infectivity in undamaged epithelial mucosal barrier	protonated lactic acid and SCFA mediated upregulation of epithelial barrier integrity markers and block of inflammatory mediators (IL-6, IL-8, TNFα, RANTES, and MIP3α)	ectocervical, endocervical and vaginal cell lines	[41,168,169]
HSV-2	*L. crispatus*	block of viral receptor binding	adhesion; microcolony formation	vaginal epithelial cells, Vero and HeLa	[170]
	*L. brevis*	reduced cell permissivity	cell wall	Vero	[171]
HSV-1	*L. crispatus* *L. gasseri*	reduction of viral titer	cell-free supernatant	Vero and HeLa	[172]
	n.a. (pH conditions evaluation)	fusogenic glycoproteins inactivation	acidic pH	n.a.	[173,174]
	n.a. (fresh, healthy mucus evaluation)	virion trapping	cervicovaginal mucus	ex vivo	[175]

n.d.: not determined; n.a.: not applicable.

**Table 2:** Clinical approaches using *Lactobacillus*-based probiotics for the treatment of viral STIs.

Infection	Participants	Probiotic(s)	Treatment	Study Type	Main Findings	Ref
HPV-LSIL	54 women (32 years on average)	*L. casei* Shirota (Yakult)	One probiotic drink (2 × 10^9^ CFU) per day for 6 months	Prospective controlled pilot study	Improved the resolution of cytological abnormalities	[253]
BV/ vaginitis with concomitant HPV	117 women (32 on average group1; 29 on average group2)	*L. rhamnosus* BMX 54 (NORMOGIN^®^) after treatment with metronidazole for 7 days or fluconazole for 2 days	Vaginal tablet (2 × 10^4^ CFU), once a day for 10 days, once every 3 days for 20 days, then once every 5 days for 2 months (group1), or once a week for 5 months (group2)	Pilot, randomized trial	Decreased HPV-related cytological abnormalities in group 2; increased HPV clearance	[254]
HR-HPV	121 women (30–65 years)	*L. rhamnosus* GR-1 + *L. reuteri* RC-14 (U-relax ^®^)	Oral capsule (5.4 × 10^9^ CFU) once a day until HPV negativization	Randomized double-blind, placebo-controlled trial	Decreased mildly abnormal and low-quality cervical smears	[255]
HPV- ASCUS or LSIL	35 women (18–65 years)	*L. crispatus* M247	Oral, 90 days	Open, non-controlled study	Improved HPV clearance; favored shift to CST I status	[149]
HR-HPV and/or LR-HPV	160 women (30–64 years)	*L. crispatus* M247	Oral, 12 months	Pilot, randomized controlled trial	Reduced HPV-related cytological anomalies	[256]
HPV	200 women (30 years on average)	Lacidophilin + rhIFN-α2b	Vaginal capsules, two at a time, the second night after rhIFN-α2b intervention, for 4 courses of treatment	Controlled pilot study	Decreased HPV-positive	[257]
HIV	115 women (18–45 years)	*L. rhamnosus* GR-1 + *L. reuteri* RC-14	Oral capsule (2 × 10^9^ CFU) twice a day for 25 weeks	Randomized, double-blind, placebo-controlled trial	Increased CD4+ count	[258]
HIV	20 subjects (18–65 years)	*L. rhamnosus* HN001 + *B. lactis* Bi-07	Oral gel formulation (10^9^ CFU) once a day for 16 weeks	Randomized, double-blind, controlled trial	Increased beneficial gut bacteria and reduced harmful ones; increased CD4+ count; decreased IL-6 levels (a marker of HIV-infected mortality)	[259]
HIV	21 subjects (47 years on average)	*L. rhamnosus* CAN-1	One yogurt daily (10^9^ CFU/mL) for 30 days	Randomized, double-blind, controlled study	Increased CD4+ count; increased in subjective energy and ability to perform daily ability score	[260]
HIV	48 subjects (39–53 years)	*L. rhamnosus* GG (Dicoflor 60)	Oral capsule (6 × 10^9^ CFU) twice a day for 16 weeks	Prospective, clinical interventional trial	Decreased intestinal inflammation; decreased harmful gut bacteria	[261]
HIV	48 subjects (42 years on average)	*L. casei* Shirota	One probiotic drink (4 × 10^10^ CFU) per day for 12 weeks	Double-blind, placebo-controlled pilot study	No adverse effects; no differences in CD4+ counts between control and treated groups	[262]
HIV	71 subjects (50 years on average)	*L. plantarum* CECT7484 + *L. plantarum* CECT748 + *P. acidilactici* CECT7483 (i3.1probiotic formulation)	Oral powder (10^9^ CFU/sachet) once a day for 30 days	Randomized, double-blind trial	Increased CD4+/CD8+ ratio	[263]
HSV-2	60 women on the 14–16th week of pregnancy	*L. crispatus* LBV88 + *L. rhamnosus* LBV96 + *L. gasseri* LBC150 + *L. jensenii* LBV116 (Florium)	Oral capsule (2.5 × 10^9^ CFU) twice a day for one week	Unspecified	Improved vaginal microbiota and vaginal discomfort; reduced complications related to pregnancy	[264]
HSV-2	81 women (17–57 years)	*L. brevis* CD2 + *L. brevis* + KB290 + *L. brevis* SBC8803	Vaginal capsule (2 × 10^9^ CFU) twice a day for 6 months	Randomized double-blind controlled trial	Reduced the healing time of the lesions and the duration of viral shedding; suppressed recurrent herpes virus infection	[265]

LSIL: low squamous intraepithelial lesions; HSIL: high squamous intraepithelial lesions; ASCUS: atypical squamous cells of undetermined significance; HR-HPV: high-risk HPV; LR-HPV: low-risk HPV.

## Data Availability

Data sharing is not applicable.
